# Notes and News

**Published:** 1892-11-26

**Authors:** 


### NOTES AND NEWS.

COLLECTED AND COMMUNICATED.

The Lord Mayor has consented to preside at the seventy-second annual court of the Seamen's Hospital Society (Dreadnought) on Wednesday, February 1st. The meeting will be held at the Mansion House.

At a drawing-room meeting in Cavendish-square, Dr. Schofield brought forward the subject of special instruction in hygiene for women. Believing, as he does, that such instruction is especially necessary, he is getting up a petition to Oxford and Cambridge to urge these Universities to make hygiene an optional subject at their higher girls and intermediate women's examinations. He himself holds a course in hygiene at the Polytechnic, the opening lecture of which, held on the 10th ult., was free to all.

The Earl of Rosebery, President of the Royal National Hospital for Consumption, Ventnor, writes to us: "Under the system of construction adopted at the Ventnor Hospital, a separate bed-room is assigned to every patient. By mechanical means a complete and frequent change of air is secured in every sitting-room and bed-room occupied by patients. Bearing in mind the recent inquiries into the cause of consumption, among other diseases, the great importance of these precautions must be evident. Dr. Hassall stongly urged the advantages of this system, and in fact founded this hospital twenty-four years ago to secure its adoption. The experience thus gained has fully confirmed the belief that pure and fresh air, with wholesome food, are among the most important matters in the treatment of this terrible complaint. Consumption probably causes more deaths in England than any other single disease. It becomes daily more difficult to obtain the necessary pecuniary support to secure the maintenance in unimpaired efficiency of hospitals. Amidst all the claims upon public benevolence none can appeal more strongly than does the Ventnor Hospital to all blessed with good health, and the means wherewith to help others not so blessed. In the hope that many of your readers may be persuaded to become annual subscribers or otherwise to contribute to our funds, I ought to add, that the payment of thirty guineas (which may be extended over three years) will entitle the donor for life to the privilege of annually recommending a patient for admission."

The question of adulterated milk was brought before the last quarterly Court of the Addenbrooke's Hospital, and it was stated that, owing to the demand for extra milk during one period, the contractor had had to purchase the required quantity elsewhere, hence the addition of water. We are unacquainted with the price given for milk at the Addenbrooke, but we may mention a fact that affects many other institutions, at any rate in this connection. A large dairy proprietor recently informed us that he did not care to undertake contracts for hospitals, as the rate paid by them was too small to make it possible to supply pure milk, and milk vendors of good standing could not afford to sell adulterated milk. Milk is so important an article of consumption in sickness that no pains should be spared to procure it really good for our hospitals, quality rather than economy should be studied in this case.

It must be admitted that the Oral system of instruction for the deaf and dumb has great advantages over any other. The association formed to promote the adoption of the system are conferring immense benefits on those so unfortunately afflicted. The foundress of the association, Baroness Meyer de Rothschild, was so convinced of the advantages of the method, as proved by experience in her own schools at the Jews' Deaf and Dumb Home, that she was induced to extend the sphere of usefulness. Since 1870 the school and college for Oral teaching has been established in Fitzroy Square. Great success has crowned the attempt, and through meetings and conferences, at which practical demonstrations of the result of the teaching were given, the public have become convinced of the advantages afforded by this system. The undertaking i not entirely self-supporting, and is one that justly appeals to our generosity. The training college for teachers, in connection with the school, is an excellent institution, and were the fact more widely known that a dearth of male teachers especially exists, it would, no doubt, be more largely patronised.

There are fashions in maladies as well as in dress, and frequently the maladies are as little new as the "latest novelty" in dress, only they are both new to our attention, and therefore of special importance. We are inclined to think that in the matter of disease, "nerves" are especially absorbing attention at the present time, and are considered quite a feature of the present age. We ourselves have cause to believe that modern nerves are very much more like their older brethren. We have only to open the pages of the lighter literature of less than a century ago, to read, with a mixture of amusement and contempt, of the "vapours," "swoons," and "sobbings," of the female element of society, and of the nervous irritability of the stronger sex. Under the light of our present knowledge, we trace the prevalence of neurotic and hysterical conditions quite unconsciously placed before us, and treated very much as a matter of course. Bearing in mind that we live in an age of pressure and hurry, that nervous tendencies are detected and classified in an unhesitating and relentless fashion, quite unknown when "vapours" and "swooning" seemed to have been the correct characteristics of "truly lady-like beings," and the broken head of a post-boy was but a mild indication of irritation on the part of young men, we consider that modern minds may with reason abate some of their "nervous" fears as to the degeneracy of the age.

